# Supplementary material for: Natural rhythms of periodic temporal attention
Source: Nat Commun. 2020 Feb 26;11:1051. doi: 10.1038/s41467-020-14888-8 (PMC7044316; doi:10.1038/s41467-020-14888-8)
Supplement: Supplementary file 1 — Supplementary Information [file 41467_2020_14888_MOESM1_ESM.pdf]

Supplementary Information

Natural rhythms of periodic temporal attention

Zalta et al.

Supplementary Figures

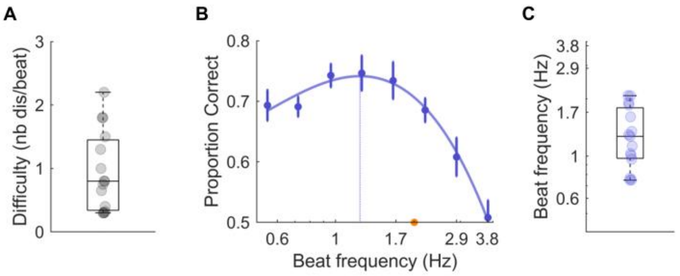

Supplementary figure 1 | Experiment 6. In this alternate version of experiment 1, the duration of the pure tones was kept constant across conditions and equal to 22.5ms. **A**. Individual difficulty level to reach threshold performance for a 2 Hz tempo. **B**. Average performance per condition. Same conventions as in Figure 1C. **C**. Individual estimates of the optimal tempo. Error bars indicate s.e.m. (n = 15). Boxplots represent median and 1.5 times the interquartile range.

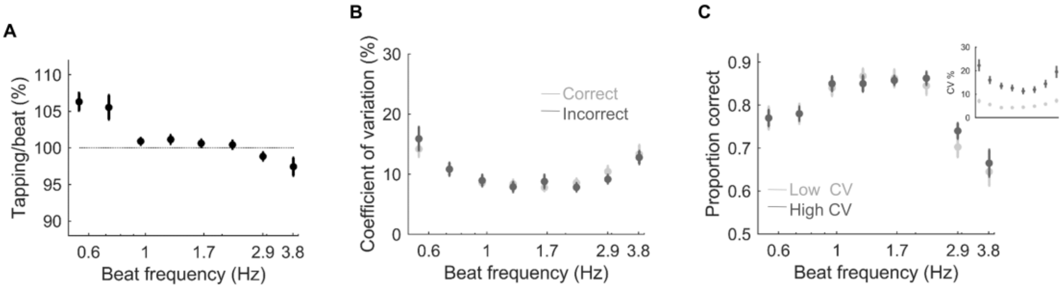

Supplementary figure 2 | Experiment 2 (auditory). **A**. Guided tapping precision across conditions of the tracking session. The precision is expressed as the ratio (in %) between the average tapping frequency and the tempo. In other words, it indicates whether the tempo of the tapping is faster (>100 %) or slower (<100 %) than the tempo of the sequence. The horizontal line indicates the ideal ratio. **B.** Coefficient of variation (CV) of guided tapping across conditions in the tracking session, for correct (light grey) and incorrect (dark grey) trials. **C**. Average performance per condition in the tracking session, for trials with low (light grey)) and high (dark grey) CV. Trials were sorted according to a median-split procedure. The inset plot indicates the associated CV. Error bars indicate s.e.m. (n = 20).

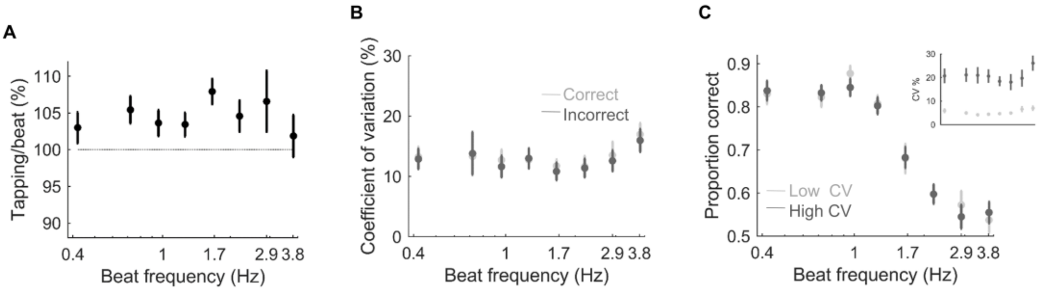

Supplementary figure 3 | Experiment 5 (visual). **A**. Guided tapping precision across conditions of the tracking session. The precision is expressed as the ratio (in %) between the average tapping frequency and the tempo. In other words, it indicates whether the tempo of the tapping is faster (>100 %) or slower (<100 %) than the tempo of the sequence. The horizontal line indicates the ideal ratio. **B.** Coefficient of variation (CV) of guided tapping across conditions in the tracking session, for correct (light grey) and incorrect (dark grey) trials. **C**. Average performance per condition in the tracking session, for trials with low (light grey)) and high (dark grey) CV. Trials were sorted according to a median-split procedure. The inset plot indicates the associated CV. Error bars indicate s.e.m. (n = 20).

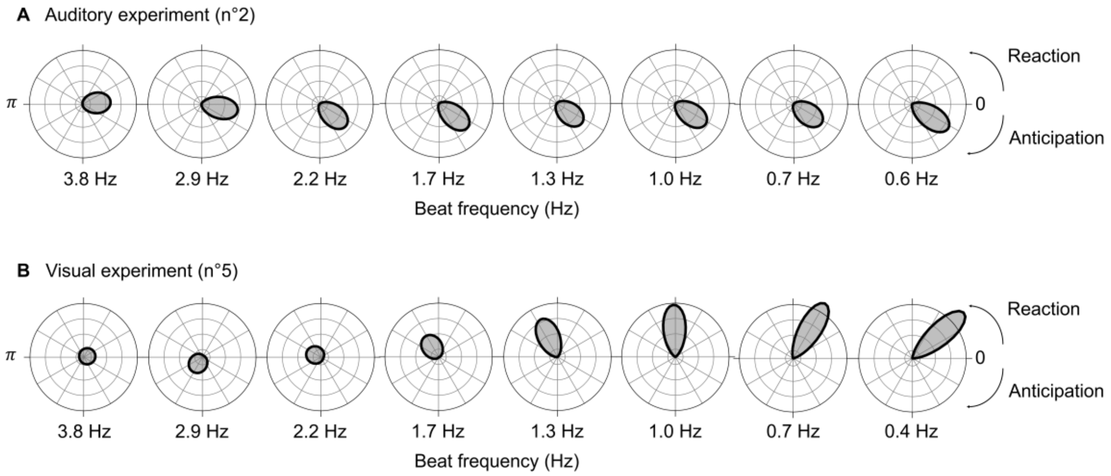

Supplementary figure 4 | Inter-individual distribution of the temporal distance between motor acts and the beat (in *relative* value, normalized to the tempo period) across conditions in the tracking session of the **(A)** auditory (exp. 2) and **(B)** visual (exp. 5) experiments. Negative (/positive) values indicate that motor acts anticipated (lagged) the beat (0: in-phase, π: antiphase).
