# Supplementary material for: Natural rhythms of periodic temporal attention
Source: Nat Commun. 2020 Feb 26;11:1051. doi: 10.1038/s41467-020-14888-8 (PMC7044316; doi:10.1038/s41467-020-14888-8)
Supplement: Supplementary file 3 — Reporting Summary [file 41467_2020_14888_MOESM3_ESM.pdf]

nature research

Corresponding author(s): Benjamin Morillon

Last updated by author(s): Nov 20, 2019

Reporting Summary

Nature Research wishes to improve the reproducibility of the work that we publish. This form provides structure for consistency and transparency in reporting. For further information on Nature Research policies, see Authors & Referees and the Editorial Policy Checklist .

Statistics

For all statistical analyses, confirm that the following items are present in the figure legend, table legend, main text, or Methods section.

n/a	Confirmed	
☐	☒	The exact sample size (*n*) for each experimental group/condition, given as a discrete number and unit of measurement
☐	☒	A statement on whether measurements were taken from distinct samples or whether the same sample was measured repeatedly
☐	☒	The statistical test(s) used AND whether they are one- or two-sided<br>*Only common tests should be described solely by name; describe more complex techniques in the Methods section.*
☐	☒	A description of all covariates tested
☐	☒	A description of any assumptions or corrections, such as tests of normality and adjustment for multiple comparisons
☐	☒	A full description of the statistical parameters including central tendency (e.g. means) or other basic estimates (e.g. regression coefficient) AND variation (e.g. standard deviation) or associated estimates of uncertainty (e.g. confidence intervals)
☐	☒	For null hypothesis testing, the test statistic (e.g. *F*, *t*, *r*) with confidence intervals, effect sizes, degrees of freedom and *P* value noted<br>*Give P values as exact values whenever suitable.*
☒	☐	For Bayesian analysis, information on the choice of priors and Markov chain Monte Carlo settings
☐	☒	For hierarchical and complex designs, identification of the appropriate level for tests and full reporting of outcomes
☐	☒	Estimates of effect sizes (e.g. Cohen's *d*, Pearson's *r*), indicating how they were calculated

Our web collection on statistics for biologists contains articles on many of the points above.

Software and code

Policy information about availability of computer code

Data collection	Behavioral responses were collected using Matlab.
Data analysis	Data analyses were performed using custom scripts in Matlab, available on Github: github.com/DCP-INS/TempAtt

For manuscripts utilizing custom algorithms or software that are central to the research but not yet described in published literature, software must be made available to editors/reviewers. We strongly encourage code deposition in a community repository (e.g. GitHub). See the Nature Research guidelines for submitting code & software for further information.

Data

Policy information about availability of data

All manuscripts must include a data availability statement. This statement should provide the following information, where applicable:

- Accession codes, unique identifiers, or web links for publicly available datasets
- A list of figures that have associated raw data
- A description of any restrictions on data availability

The entire dataset of this study and codes to reproduce the results and figures of this manuscript are available on GitHub: https://github.com/DCP-INS/TempAtt.

Field-specific reporting

Please select the one below that is the best fit for your research. If you are not sure, read the appropriate sections before making your selection.

☐ Life sciences ☒ Behavioural & social sciences ☐ Ecological, evolutionary & environmental sciences

For a reference copy of the document with all sections, see nature.com/documents/nr-reporting-summary-flat.pdf

Behavioural & social sciences study design

All studies must disclose on these points even when the disclosure is negative.

Study description	Quantitative psychophysical auditory or visual discrimination (yes/no) tasks, derived from a previous study (Morillon et al., 2014).
Research sample	30, 20, 50, 30, 20 and 15 participants (age range: 18–35 years; 69% of females) were respectively recruited for experiments 1 to 6. The samples chosen are representative of the general population and where chosen on the basis of their willigness to participate to the study and their availability at the moment of data collection.
Sampling strategy	Random sampling and quota sampling. Sample size estimation based on previous studies (Morillon et al., 2014; Morillon and Baillet, 2017).
Data collection	Data were collected with computers during experimental laboratory sessions.
Timing	Experiment 1 : 28 june, 2018 - 10 september, 2018<br>Experiment 2 : 17 september, 2018 - 12 dicember, 2018<br>Experiment 3 : 15 september, 2018 - 15 dicember, 2018<br>Experiment 4 : 9 august, 2018 - 15 november, 2018<br>Experiment 5 : 4 dicember, 2018 - 14 dicember, 2018<br>Experiment 6 : 3 january, 2019 - 29 january, 2019<br>During data collection no one was present besides the participant and the experimentator. This latter was blind to the experimental conditions.
Data exclusions	All data exclusions are reported in the manuscript; we also provide analyses towards that end (that is, we report analyses of both the full sample and the reduced sample based on pre-established filtering criteria/attention checks)
Non-participation	No participants declined to participate.
Randomization	Participants were randomly assigned to experimental conditions beginning.

Reporting for specific materials, systems and methods

We require information from authors about some types of materials, experimental systems and methods used in many studies. Here, indicate whether each material, system or method listed is relevant to your study. If you are not sure if a list item applies to your research, read the appropriate section before selecting a response.

Materials & experimental systems

n/a	Involved in the study	
☒	☐	Antibodies
☒	☐	Eukaryotic cell lines
☒	☐	Palaeontology
☒	☐	Animals and other organisms
☐	☒	Human research participants
☒	☐	Clinical data

Methods

n/a	Involved in the study	
☒	☐	ChIP-seq
☒	☐	Flow cytometry
☒	☐	MRI-based neuroimaging

Human research participants

Policy information about studies involving human research participants

Population characteristics	All participants were healthy volunteers. All participants were fluent in the French language.<br>(see above for age and gender characteristics)
Recruitment	Adverts on campus and online, e-mailing campaigns, direct requests, and contacting facebook groups.<br>No obvious biases regarding the population recruited.
Ethics oversight	The institutional review board at Aix Marseille University approved the study protocol.

Note that full information on the approval of the study protocol must also be provided in the manuscript.
